# Immunohistochemical expression of GPNMB in *TFEB*-rearranged and *TFEB*-amplified renal cell carcinomas

**DOI:** 10.1007/s00428-026-04466-6

**Published:** 2026-03-04

**Authors:** Kristyna Pivovarcikova, Petr Grossmann, Petr Steiner, Levente Kuthi, Joanna Rogala, Kiril Trpkov, Jose Ignacio Lopez, Boris Rychly, Lucia Sarvaicova, Zuzana Spurkova, Ondrej Nikolov, Ales Mlynek, Jiri Soukup, Eva Sehnalkova, Ludek Baumbruk, Josef Skopal, Petr Stransky, Adriena Bartos-Vesela, Tomas Pitra, Milan Hora, Michal Michal, Ondrej Ondic, Reza Alaghehbandan

**Affiliations:** 1 Šikl’s Department of Pathology, Faculty of Medicine and University Hospital in Pilsen, Charles University, Pilsen, Czech Republic; 2 Department of Surgical and Molecular Pathology, Tumor Pathology Center, National Institute of Oncology, Budapest, Hungary; 3 Department of Pathology and Experimental Cancer Research, Semmelweis University, Budapest, Hungary; 4 HUN‐REN‐ONKOL‐TTK‐HCEMM Oncogenomics Research Group, National Institute of Oncology, Budapest, Hungary; 5 Department of Pathology, Regional Specialist Hospital, Wroclaw, Wroclaw, Poland; 6 Diagnostic and Molecular Pathology, Alberta Precision Laboratories and University of Calgary, Calgary, AB Canada; 7 Biobizkaia Health Research Institute, Barakaldo, Spain; 8 Diagnostic Pathology Centre, Unilabs Slovakia Ltd, Bratislava, Slovakia; 9 Department of Pathology, Faculty Hospital, Trnava, Slovakia; 10 Department of Pathology, Bulovka University Hospital, Prague, Czech Republic; 11 Department of Pathology, Hospital Ceske Budejovice, Ceske Budejovice, Czech Republic; 12 Department of Pathology, City Hospital Ostrava, Ostrava, Czech Republic; 13 Department of Pathology, Military University Hospital, Prague, Czech Republic; 14 Department of Pathology, First Faculty of Medicine and General University Hospital in Prague, Charles University, Prague, Czech Republic; 15 The Fingerland Department of Pathology, Faculty of Medicine in Hradec Králové and University Hospital, Charles University, Hradec Kralove, Czech Republic; 16 Department of Pathology, Silesian Hospital, Opava, Czech Republic; 17 Department of Pathology, Regional Hospital Pribram a.S., Příbram, Czech Republic; 18 Department of Urology, Faculty of Medicine and University Hospital in Pilsen, Charles University, Pilsen, Czech Republic; 19 Robert J. Tomsich Pathology and Laboratory Medicine Institute, Department of Anatomic Pathology, Cleveland Clinic, Cleveland, OH USA; 20 Department of Pathology, Medical Faculty and Charles University Hospital Plzen, Alej Svobody 80, 323 00 Pilsen, Czech Republic

**Keywords:** *TFEB*-altered renal cell carcinoma, *TFEB*-amplified renal cell carcinoma, *TFEB*-rearranged renal cell carcinoma, GPNMB, Immunohistochemistry

## Abstract

*TFEB*-altered renal cell carcinomas (RCCs) display a wide range of morphological features and variable immunohistochemical profiles, often overlapping with other RCC subtypes. This variability poses significant diagnostic challenges. Consequently, there is an ongoing search for reliable ancillary tests that can aid in identifying cases that warrant molecular testing. In this study, we focused on GPNMB immunohistochemistry expression in *TFEB*-rearranged and *TFEB*-amplified RCCs. A total of 25 *TFEB*-altered RCCs, including 16 *TFEB*-amplified RCCs and nine *TFEB*-rearranged RCCs, were included in the study. GPNMB immunohistochemistry was positive in all nine *TFEB*-rearranged RCCs, demonstrating strong, diffuse cytoplasmic staining. In the *TFEB*-amplified RCC cohort, GPNMB expression was also detected in all 16 tumors; however, the staining pattern differed from that seen in *TFEB*-rearranged RCCs. Diffuse staining was observed in eight tumors (50%), four of which showed variable staining intensity, while the remaining eight tumors (50%) exhibited focal GPNMB reactivity, with intervening negative areas. GPNMB was therefore positive in all 25/25 (100%) *TFEB*-altered RCCs by IHC, supporting its potential role as a sensitive screening marker for such RCCs. *TFEB*-rearranged RCCs typically exhibited strong, diffuse GPNMB immunoreactivity, whereas *TFEB*-amplified RCCs more often showed patchy or focal staining of lower intensity, interspersed with areas of GPNMB negativity. Variability of staining in *TFEB*-amplified RCCs underscores the need for cautious interpretation, particularly in limited biopsy specimens, taking into account the morphologic findings and other immunohistochemistry stains, such as Melan A and Cathepsin K.

**Supplementary Information:**

The online version contains supplementary material available at https://doi.org/10.1007/s00428-026-04466-6.

## Introduction

*TFEB*-altered renal cell carcinoma (RCC) is an umbrella term for RCCs that either harbor gene fusions involving the *TFEB* transcription factor (*TFEB*-rearranged RCCs) or exhibit amplification of the 6p21 locus containing *TFEB*, leading to its overexpression (*TFEB*-amplified RCCs). The term was introduced by the Genitourinary Pathology Society (GUPS) in 2021 [1] and it was later adopted by the World Health Organization (WHO) 2022 classification of renal cell tumors (5th Edition) [2]. *TFEB*-altered RCCs display a wide range of morphological features and immunohistochemical profiles, often overlapping with other RCC subtypes. This variability poses significant diagnostic challenges in routine practice. Consequently, there is an ongoing need for reliable ancillary tests to aid in identifying cases that warrant molecular testing.

Glycoprotein nonmetastatic melanoma protein B (GPNMB) has been reported as a sensitive immunohistochemical screening marker for *TFEB*- and *TFE3*-altered RCCs, as well as for several renal tumor types demonstrating *TSC1*, *TSC2*, or *MTOR* mutations [3–5]. However, in one study, false-negative or equivocal GPNMB results were observed in 13% of FISH-confirmed *TFE3*/*TFEB* altered cases [5]. In the current study, we examined the GPNMB expression by immunohistochemistry (IHC) in a multi-institutional cohort of *TFEB*-altered RCCs, including both *TFEB*-rearranged and *TFEB*-amplified tumors that were confirmed by molecular evaluation and/or fluorescence in situ hybridization (FISH).

## Materials and methods

### Case selection

The Plzen Tumor Registry (counting more than 10,000 renal tumors) was searched for *TFEB*-altered RCCs (*TFEB*-rearranged RCC and *TFEB*-amplified RCC), using the following keywords: Kidney, Renal Cell Carcinoma, TFEB. Samples were used in accordance with the ethical guidelines. Informed consent was not required for the study.

We included all *TFEB*-altered RCCs, confirmed by FISH. As for *TFEB*-amplified RCCs, we only included “high amplified” tumors, characterized by an amplified signal ≥ 10:1 ratio of *TFEB* signal [6], as summarized in Suppl. Table 1.

Tissue for light microscopy was fixed in 4% formaldehyde and embedded in paraffin using the routine procedure. Sections 2-µm thick were cut from tissue blocks and stained with hematoxylin and eosin (H&E).

### Immunohistochemistry

A representative tumor block was stained for GPNMB (E4D7P, monoclonal, Cell Signaling Technology; 1:500); the cytoplasmic staining results for GPNMB were evaluated. Further, we also performed IHC evaluation by a panel of antibodies that included AE1/3 (AE1/AE3/PCK26, monoclonal; Ventana Medical Systems; RTU), vimentin (V9, monoclonal, Ventana Medical Systems; RTU), melan A (A 103, monoclonal; Agilent Dako; 1:50), HMB45 (HMB45, monoclonal; Agilent Dako; 1:200), cathepsin K (3F9, monoclonal, Abcam; 1:300), CK7 (OV-TL12/30, monoclonal; Agilent Dako; 1:100), CK20 (Ks20.8, monoclonal; Agilent Dako; 1:50), CD117 (polyclonal, Agilent Dako; 1:500), PAX8 (MRQ-50, monoclonal, Cell Marque; RTU), TFE3 (MRQ-37, monoclonal, Cell Marque; RTU), FH (J-13, monoclonal, Santa Cruz Biotechnology, 1:3000), and anti-SDHB (polyclonal; Sigma Aldrich; 1:200). IHC staining was performed using Ventana Benchmark XT automated stainer (Ventana Medical System, Inc., Tucson, AZ, USA). Appropriate positive controls were used for all stains and slides. According to the percentage of tumorous mass positive, immunohistochemical staining was classified as follows: negative (no positive cells/-), single cells positive (SC), focal/patchy positive (≥ 1% and < 50% positive cells), and diffusely positive (≥ 50% positive cells). According to the intensity of staining, the stain was evaluated as follows: weakly positive (+), moderately positive (+ +), strongly positive (+ + +).

## Results

A total of 25 *TFEB*-altered RCCs, including 16 *TFEB*-amplified RCCs and nine *TFEB*-rearranged RCCs, were included in the study. Clinicopathologic data are summarized in Supplementary Table 2. Patients were 12 males and 13 females, ranging in age from 11 to 85 years (mean 62.5; median 73 years). Mean tumor size was 6 cm (range 2.2–13; median 4.5 cm).

### TFEB-rearranged RCC

#### Clinicopathologic and microscopic findings

*TFEB*-rearranged RCC patients were one male and eight females, ranging in age from 11 to 73 years (mean 41.4; median 39 years). Mean tumor size was 6.3 cm (range 3–10; median 5.8 cm).

**Fig. 1:**
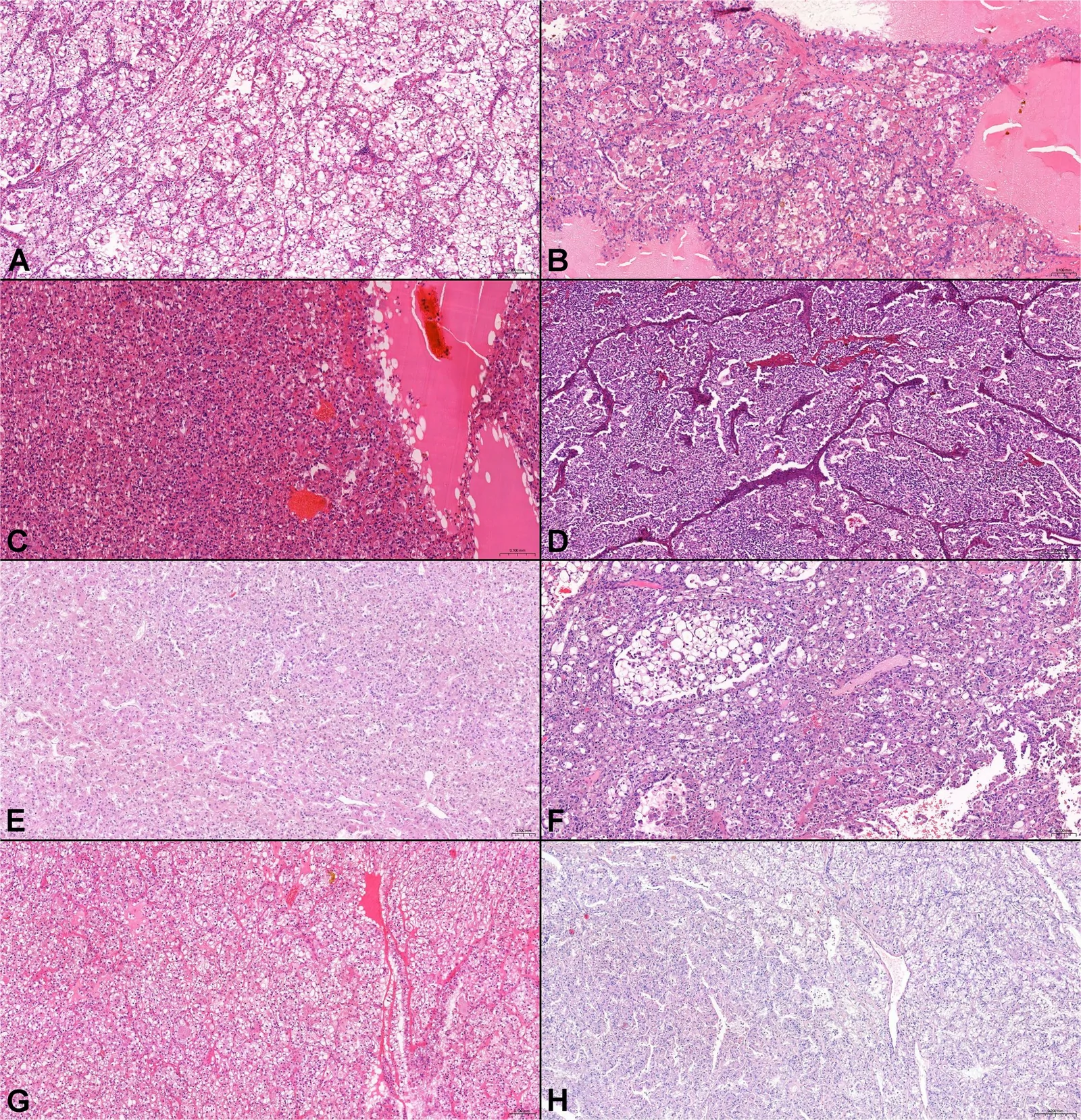
The morphologic spectrum of *TFEB*-rearranged RCCs was broad. Confluent and solid growth patterns predominated, occasionally in combination with other growth patterns (acinar, papillary, cystic). Tumor cells exhibited at least focal, finely eosinophilic granular cytoplasm, while admixed clear cells were present in a subset of tumors (55.6%). Representative areas of some *TFEB*-rearranged RCCs are shown: case 1 (**A**), case 2 (**B**), case 3 (**C**), case 4 (**D**), case 5 (**E**), case 6 (**F**), case 7 (**G**), and case 8 (**H**)

Microscopically, *TFEB*-rearranged RCCs showed a broad morphologic spectrum. Confluent and solid growth patterns predominated (66.7%), occasionally in combination with acinar, papillary, or cystic architectures. In all cases, tumor cells exhibited at least focal, finely eosinophilic granular cytoplasm, while admixed clear cells were present in a subset of tumors (five cases; 55.6%). Seven of nine tumors (77.8%) were WHO/ISUP grade 3, and two (22.2%) were grade 2. None of the tumors showed calcifications, necrosis, or sarcomatoid/rhabdoid differentiation. The morphologic spectrum of the tumors is shown in Fig. 1.

#### Immunohistochemical findings

**Fig. 2:**
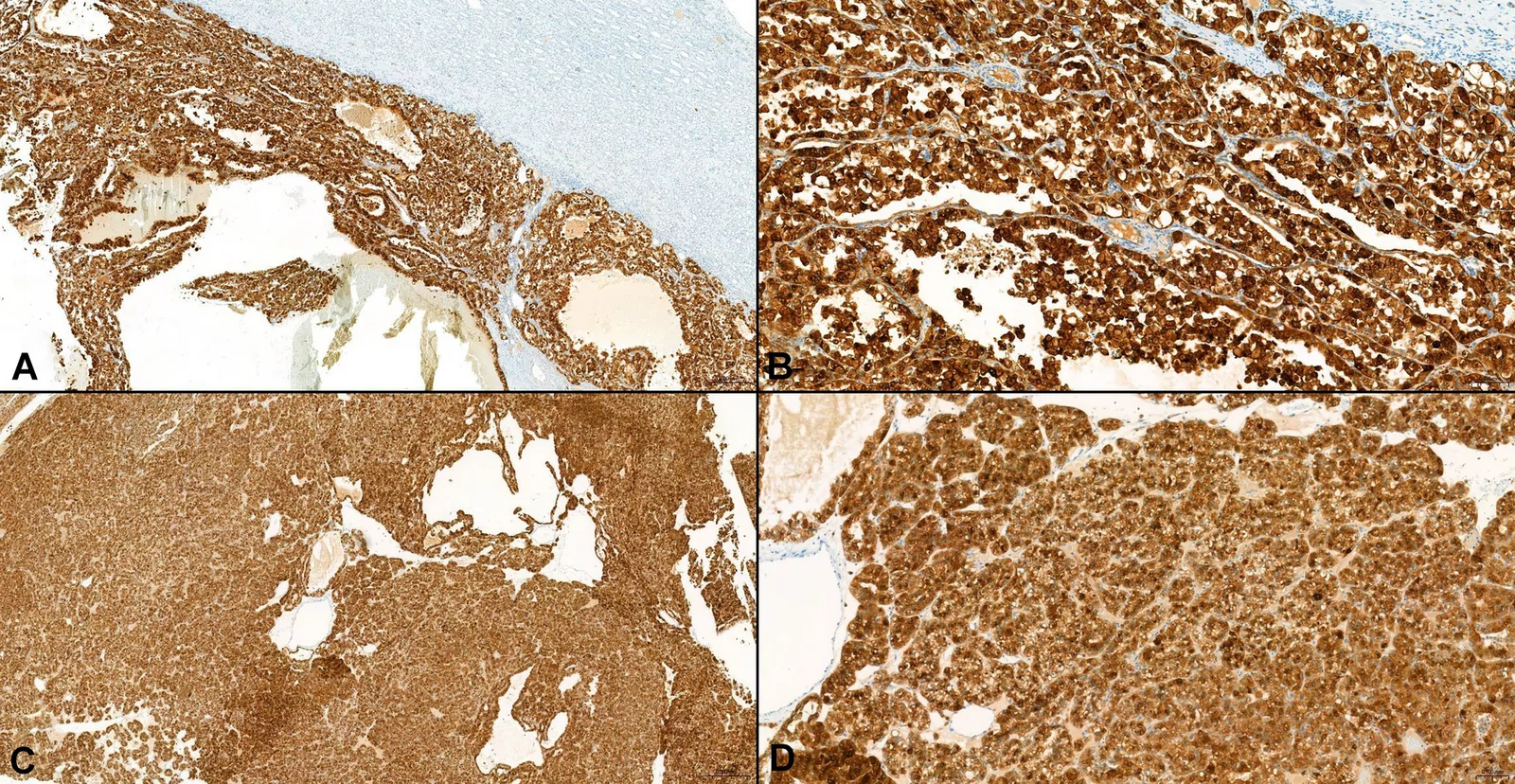
In *TFEB*-rearranged RCCs, GPNMB was positive in all tumors in strong and diffuse fashion —case 2 (**A**, **B**), case 5 (**C**, **D**)

A summary of the IHC findings is provided in Table 1. Eight of nine tumors showed reactivity for one or two traditional melanocytic markers (HMB45 in seven of nine cases and melan A in eight of nine cases), at least in scattered cells. Cathepsin K was positive in all tumors. Pancytokeratin AE1/3 expression was identified in only two of nine cases (focal in one). CD117 reactivity was observed in four of nine tumors, while PAX8 was negative in three of nine cases. SDHB and FH were retained in all *TFEB*-rearranged RCCs. CK20 expression was detected in three of nine tumors, with only single cell staining in two. GPNMB was positive in all tumors, showing strong and diffuse staining (Fig. 2).

**Table 1:** Immunohistochemistry findings in *TFEB*-rearranged and *TFEB*-amplified RCCs

Case	HMB45	Melan A	AE1/3	CD117	Cathepsin K	TFE3	PAX 8	CK7	FH	SDHB	CK20	GPNMB
*TFEB*-rearranged RCC												
Case 1	Dif/+ +	Foc/+ + +	-	-	Dif/+ +	Foc/+	Foc/+	-	Retained	Retained	-	Dif/+ + +
Case 2	Foc/+ +	Foc/+	Foc/+ + +	-	Dif/+ +	-	Foc/+	SC/+ +	Retained	Retained	-	Dif/+ + +
Case 3	Foc/+ +	Foc/+	-	-	Dif/+	-	-	-	Retained	Retained	-	Dif/+ +
Case 4	Foc/+	Dif/+ +	-	Foc/+ +	Dif/+ + +	-	Foc/+	-	Retained	Retained	-	Dif/+ + +
Case 5	-	Foc/+ +	-	Foc/+ +	Dif/+ + +	-	Foc/+	-	Retained	Retained	-	Dif/+ + +
Case 6	SC/+ + +	Foc/+ +	-	-	Dif/+ +	-	Foc/+ +	-	Retained	Retained	SC/+ + +	Dif/+ + +
Case 7	SC/+ +	Dif/+ +	-	Dif/+ +	Dif/+ +	-	Foc/+ +	-	Retained	Retained	SC/+ +	Dif/+ + +
Case 8	Foc/+	Dif/+ + +	-	-	Dif/+ + +	-	-	-	Retained	Retained	-	Dif/+ + +
Case 9	-	-	Dif/+ +	Dif/+	Dif/+ + +	-	-	-	Retained	Retained	Dif/+ +	Dif/+ + +
*TFEB*-amplified RCCs												
Case 10	-	-	Foc/+ + +	-	Dif/+ +	-	Dif/+ + +	Dif/+ + +	Retained	Retained	SC/+ + +	Foc/+ + + (irregular)
Case 11	Dif/+	Foc/+ +	-	-	Foc/+ + +	-	Foc/+	-	Retained	Retained	Foc/+ + +	Foc/+ + + (irregular)
Case 12	-	Foc/+ + +	-	-	Dif/+ + +	-	Foc/+ + +	-	Retained	Retained	SC/+ + +	Foc/+ + (irregular)
Case 13	-	-	Foc/+ + +	-	Dif/+	Foc/+	Dif/+ +	Foc/+ + +	Retained	Retained	SC/+ +	Foc/+ + (irregular)
Case 14	-	Foc/+ +	-	-	-	-	-	Foc/+ +	Retained	Retained	SC/+	Dif/+ + +
Case 15	-	-	Foc/+	-	Foc/+	-	Dif/+	SC/+ + +	Retained	Retained	Foc/+ + +	Foc/+ + + (irregular)
Case 16	-	Foc/+ +	-	-	Dif/+ + +	-	Foc/+ +	-	Retained	Retained	-	Dif/+ + +
Case 17	-	Foc/+ + +	Dif/+ + +	-	Dif/+ +	-	NA	Foc/+ + +	Retained	Retained	SC/+ + +	Dif/+ +
Case 18	-	-	Dif/+ + +	-	Dif/+	-	Dif/+	Dif/+	Retained	Retained	-	Foc/+ + (irregular)
Case 19	-	SC/+	Dif/+ +	-	Dif/+ +	-	Dif/+ +	-	Retained	Retained	Dif/+ + +	Dif/+ + + (irregular)
Case 20	-	-	Dif/+ + +	-	Dif/+	-	Dif/+ +	-	Retained	Retained	Foc/+ + +	Foc/+ + + (irregular)
Case 21	-	SC + + +	Dif/+ + +	-	Dif/+	Dif/+	Dif/+ + +	Foc/+ + +	Retained	Retained	SC/+ + +	Foc/+ + (irregular)
Case 22	-	SC + + +	Dif/+ +	NA	Foc/+	NA	Dif/+ +	-	Retained	Retained	SC/+ + +	Dif/+ + + (irregular)
Case 23 *	-	Foc + +	Foc/+ + +	Foc/+ +	Foc/+	NA	Foc/+ +	-	Retained	Retained	SC/+ + +	Dif/+ + +
Case 24	-	SC + + +	Dif/+ + +	-	-	NA	NA	Foc/+ +	Retained	Retained	SC/+ + +	Dif/+ + + (irregular)
Case 25	-	-	Dif/+ + +	-	Dif/+	-	Dif/+	Foc/+	Retained	Retained	-	Dif/+ + + (irregular)

- negative (no positive cells), + weak positivity, ++ moderate positivity, +++ strong positivity, *Dif* diffusely positive (≥ 50% positive cells), *Foc* focal/patchy positive (≥ 1% and < 50% positive cells), *SC* single cells positive, *NA* not available

*TFEB-amplified and translocated RCC

### TFEB-amplified RCC

#### Clinicopathologic and microscopic findings

There were ten males and five females with age range of 50–85 years (mean 74.3 years; median 75 years), tumor size (available in 14/16 cases) ranged from 2.2 to 13 cm (median 4.1, mean 5.8 cm). Of note, there were no patients younger than 50 years in this group.

**Fig. 3:**
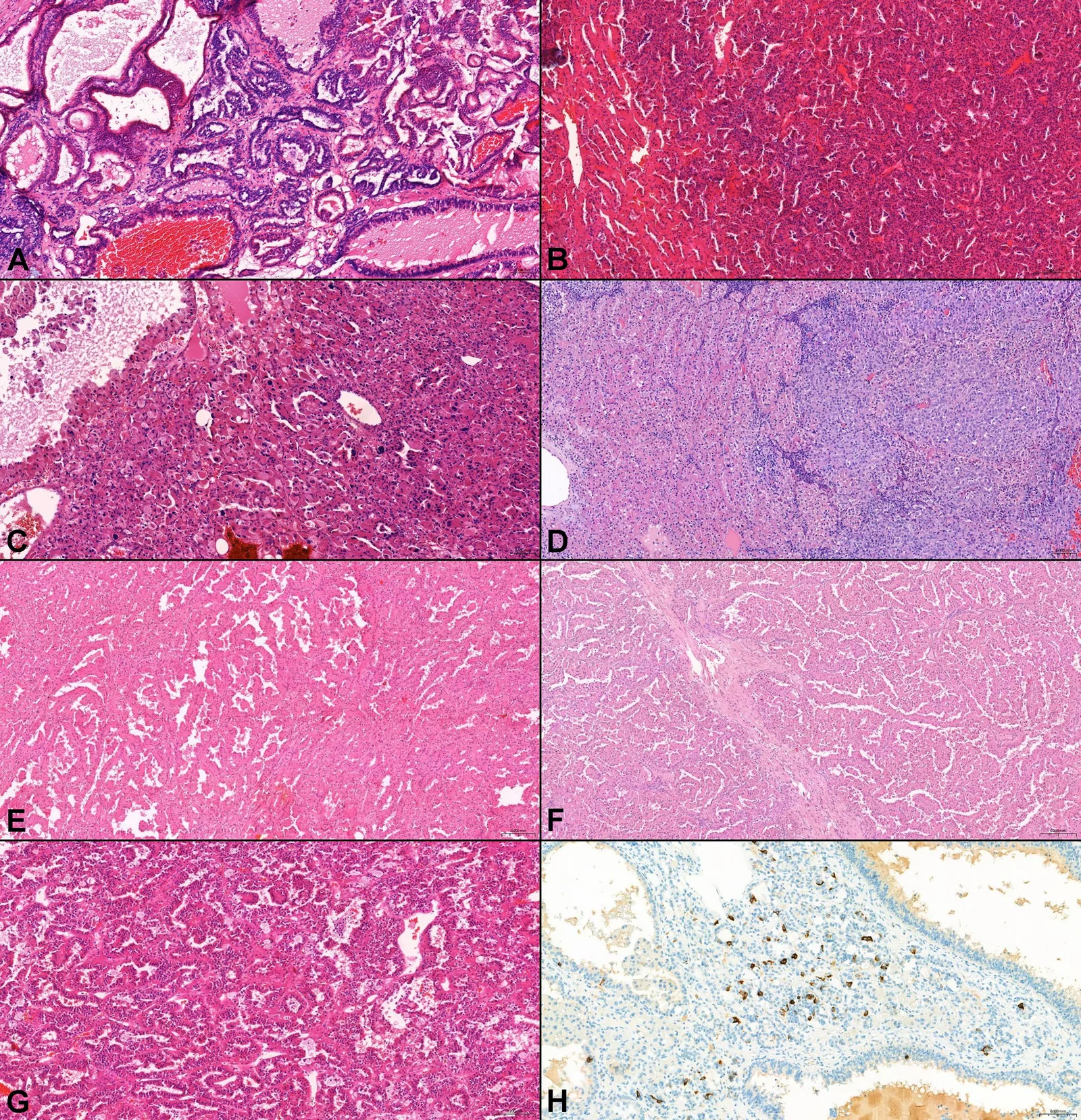
*TFEB*-amplified RCCs showed marked morphologic heterogeneity. All tumors were composed of cells with abundant granular eosinophilic cytoplasm. The predominant growth pattern was papillary/tubulopapillary, observed in 9 of 16 cases—illustrated in case 11 (**B**), case 14 (**E**), case 15 (**F**), and case 16 (**G**). Other growing patterns were as follows: tubular (case 10, **A**), cystic, confluent/solid (case 12, **C;** and case 13, **D**). CK20 reactivity was found in 13 of 16 tumors (in 12 cases, the positivity was focal or only in rare single cells (**H**))

The main morphologic patterns are shown in Fig. 3. The predominant growth pattern was papillary/tubulopapillary, observed in nine of 16 cases. Two tumors showed a predominantly tubular architecture (one with associated cystic areas and one with solid areas). Three tumors were predominantly cystic with accompanying solid components; one case demonstrated mainly cystic growth with tubulopapillary areas, and one tumor exhibited a solid and nested pattern. All tumors were composed of cells with abundant granular eosinophilic cytoplasm; focal transition to clear cell areas was identified in only three cases. Eleven tumors (68.8%) were WHO/ISUP grade 3, while five (31.2%) were grade 2. Rhabdoid/sarcomatoid morphology (grade 4) was present in one case, and calcifications were identified in six cases.

### Immunohistochemical findings

**Fig. 4:**
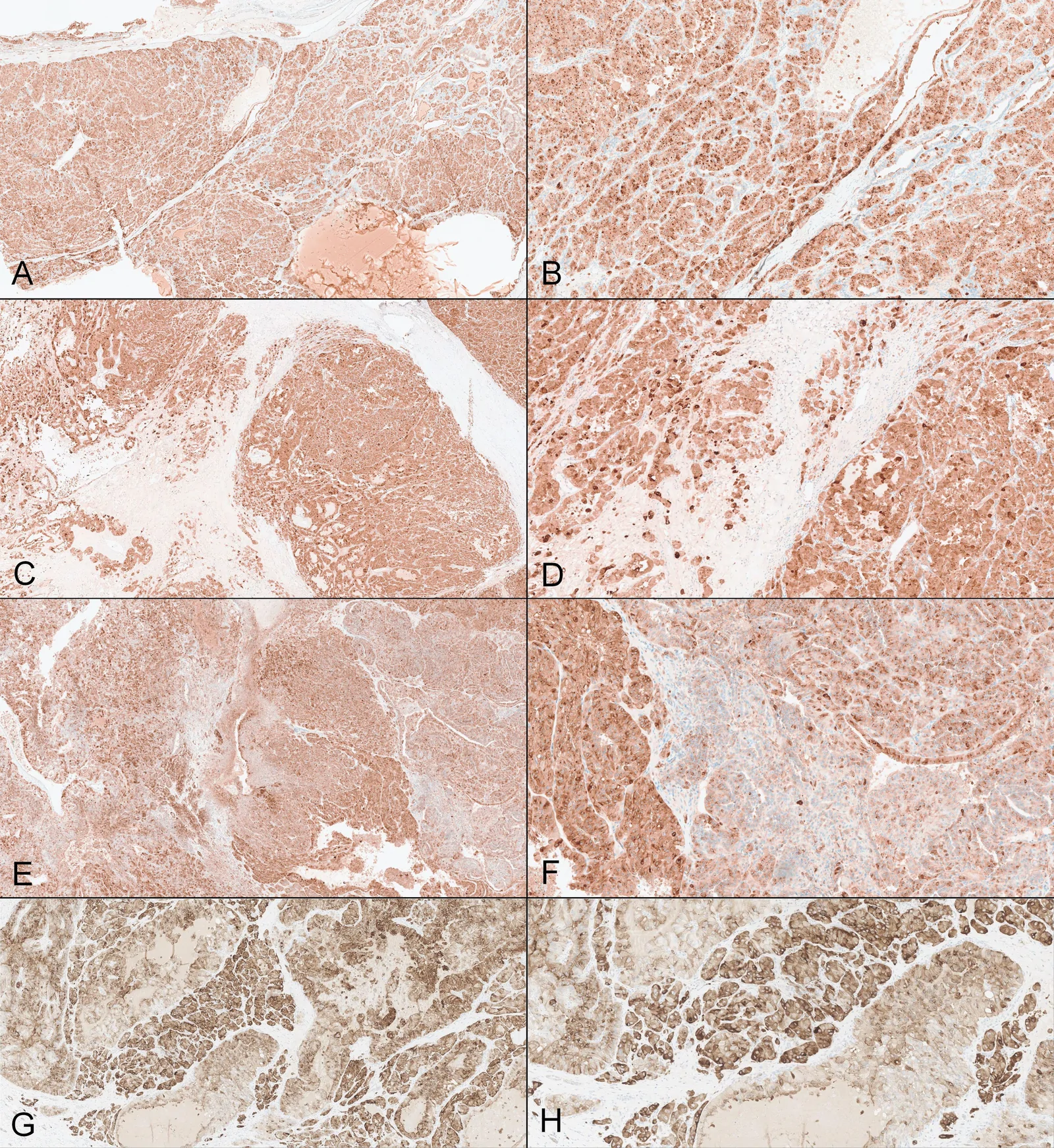
In *TFEB*-amplified RCCs, diffuse pattern of GPNMB staining was observed in 8 of 16 tumors (50%) — case 17 (**A**, **B**) and case 23 (**C**, **D**). While 4 *TFEB*-amplified RCCs with diffuse GPNMB staining exhibited marked heterogeneity with irregular staining intensity throughout the tumor, as illustrated by case 22 (**E**, **F**) and 25 (**G**, **H**)

**Fig. 5:**
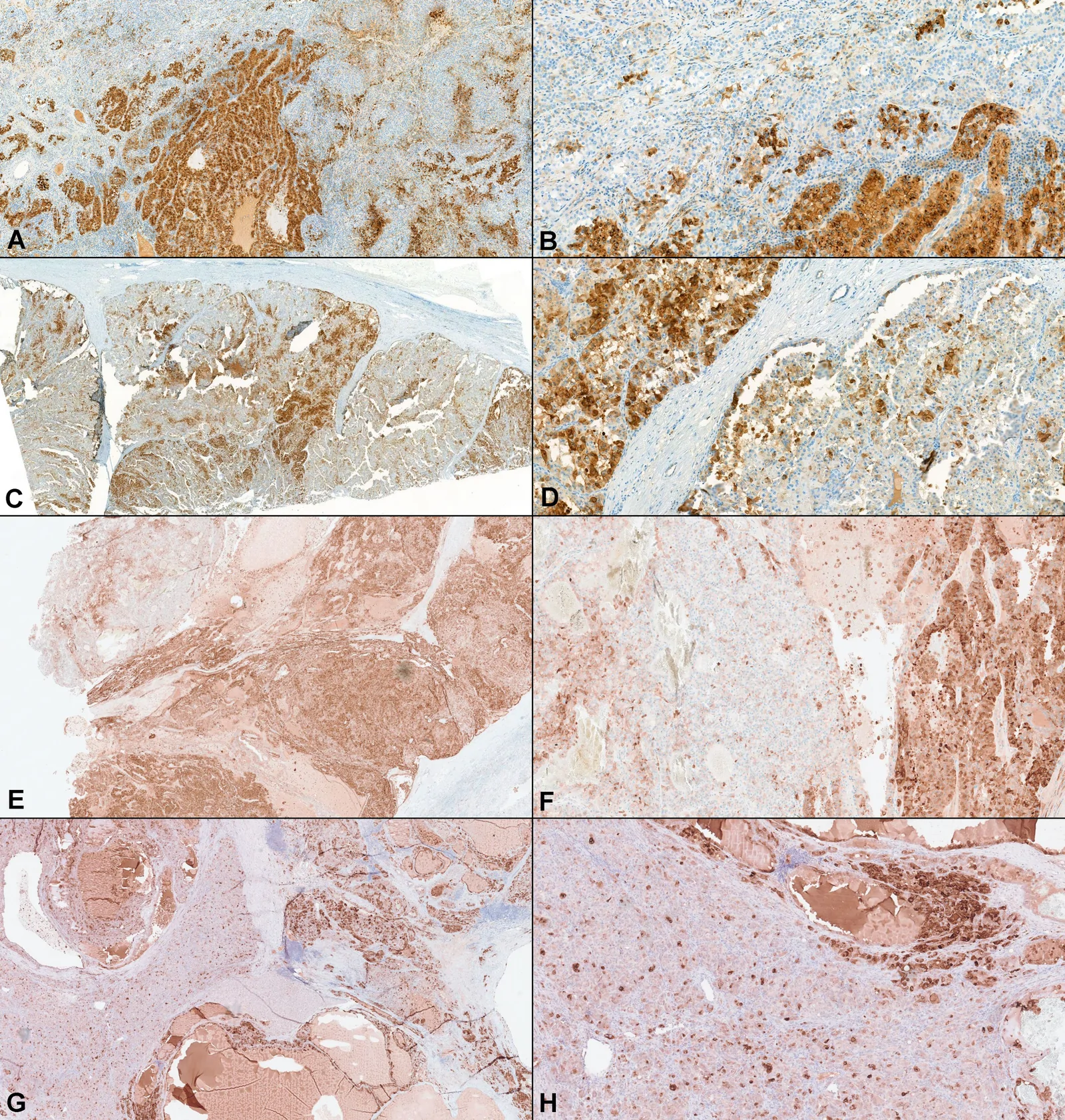
In 8 *TFEB*-amplified RCCs, GPNMB immunohistochemistry demonstrated focal reactivity, characterized by zonal distribution of intermixed negative areas with patchy labeling within the positive —case 13 (**A**, **B**), case 15 (**C**, **D**), case 20 (**E**, **F**), and case 21 (**G**, **H**)

Melan A was positive in 10/16 (62.5%) *TFEB* amplified RCCs (typically focally/or in single cells) and HMB45 was weakly expressed in only one case. Six of 16 (37.5%) tumors were completely negative for both melan A and HMB45. AE1/3 was positive in 12 of 16 (75%) tumors (in four cases focally) and CK7 was positive in nine of 16 (56.3%) cases (usually focally). Cathepsin K and PAX8 stained 14 of 16 (87.5%) and 13 of 14 (92.9%) cases, respectively. Interestingly, CK20 reactivity was found in 13 of 16 tumors (the positivity was focal or in rare single cells in 12 cases, while only one case showed diffuse strong CK20 reactivity). GPNMB was expressed in all tumors, however the staining was different than in *TFEB* rearranged RCC group. Diffuse GPNMB staining was observed in eight of 16 tumors (Fig. 4A–D), while four tumors exhibited marked heterogeneity with variable staining intensity throughout the tumor (Fig. 4E–H). In the remaining eight tumors (50%), GPNMB immunohistochemistry demonstrated focal reactivity, characterized by intermixed negative areas and irregular, patchy labeling (moderate to strong intensity) within the positive regions (Fig. 5).

## Discussion

*TFEB*-rearranged and *TFEB*-amplified RCCs are currently classified under the broader category of *TFEB*-altered RCCs [2]. In both RCC subtypes, *TFEB* gene fusion or amplification leads to overexpression of the native TFEB protein. This overexpressed TFEB functions as an aberrant transcription factor, driving the expression of numerous downstream targets, including genes typically regulated by the related microphthalmia translocation family member, MITF [2]. However, distinct clinical differences between *TFEB*-amplified RCC and *TFEB*-rearranged RCC have become increasingly evident [7].

*TFEB*-rearranged RCC was first described by Argani et al. in 2001 as a pediatric renal neoplasm characterized by distinctive epithelioid morphology, unique immunohistochemical profile, and a specific cytogenetic background [8]. *TFEB*-rearranged RCC was incorporated into the 2016 WHO Tumor Classification as a tumor characterized by distinctive biphasic morphology, occurring not only in pediatric patients but also in adults, and typically exhibiting non-aggressive behavior [9]. In 2014, Peckova et al., followed by other research groups, described RCCs with amplification of the *TFEB* gene, also leading to TFEB overexpression [7, 10–12]. Studies have shown that *TFEB*-amplified RCC primarily occurs in older patients compared to *TFEB*-rearranged RCC. Importantly, *TFEB*-amplified RCC is frequently associated with aggressive clinical behavior. The morphology of *TFEB*-amplified and *TFEB*-rearranged RCC can however overlap, as well as with other more common renal neoplasms [13], which poses diagnostic challenges in routine practice.

The IHC screening of renal tumors for translocation-type RCCs, particularly for *TFEB*-altered RCC, remains challenging, because of their rarity and the imperfections of the currently available markers. Both *TFEB*-rearranged and *TFEB*-amplified RCCs exhibit reduced expression of epithelial immunohistochemical markers (i.e., cytokeratin and epithelial membrane antigen) [13]. *TFEB*-altered RCCs typically show aberrant expression of melanocytic markers, particularly melan A, and cathepsin K. However, this expression tends to be more variable in *TFEB*-amplified RCCs compared to *TFEB*-rearranged counterparts [7] and a significant number of *TFEB*-amplified RCCs can be entirely negative for melanocytic markers, or may show limited or negative expression for cathepsin K, as supported by our findings. Recently, *TRIM63* was identified as a cancer-specific biomarker in MiTF RCC; its expression can be evaluated by RNA in situ hybridization (RNA-ISH). Studies have documented *TRIM63* RNA-ISH and *TFE3*/*TFEB* FISH results concordant [14]. Reportedly, *TRIM63* can improve the diagnostic accuracy of recognizing MiTF RCC in an adequate histologic background and distinguish them from other renal cell tumors [15]. Mannan et al. suggested that the TRIM63 RNA ISH assay could help identify cases harboring *TFE3* or *TFEB* rearrangements that are associated with false-negative or equivocal *TFE3*/*TFEB* FISH results [16].

GPNMB is a transmembrane protein expressed at low levels in most normal tissues. High levels of GPNMB expression have been reported in various tumors, including melanoma, triple-negative breast cancer, and gliomas [17]. Of note, several studies have also highlighted the utility of GPNMB IHC as an ancillary diagnostic tool in specific differential diagnoses, when evaluating a broad morphologic spectrum of tumors with underlying *TSC1/TSC2/mTOR* alterations, including perivascular epithelioid cell tumor (PEComa)/angiomyolipoma (AML) [18], low-grade oncocytic tumor (LOT) [3, 5], eosinophilic solid and cystic (ESC) RCC [3, 5], eosinophilic vacuolated tumor (EVT) [3, 5], RCC with fibromyomatous stroma (RCC FMS) [19], primary renal haemangioblastoma [20], RCC FMS with hemangioblastoma-like areas, both in a tuberous sclerosis [21] and in sporadic setting [22], subependymal giant cell astrocytoma [23], and *FLCN*-associated eosinophilic renal tumors [24]. GPNMB overexpression was noted in mouse models of *TFE3*-rearranged RCC [17], which led to the identification of GPNMB as a proposed marker for MiTF translocation RCC [25]. Salles et al. investigated GPNMB expression across a range of renal tumors, including *TFE3*- and *TFEB*-driven RCCs, RCCs associated with *TSC1*/*TSC2*/*mTOR* alterations, and a limited number of other renal neoplasms. They demonstrated GPNMB positivity in 82% of FISH-confirmed *TFE3*- and *TFEB*-altered RCCs [3]. However, it is unclear what proportion of their cases represented *TFEB*-rearranged vs *TFEB*-amplified RCCs. Whaley et al. documented GPNMB expression in 92% of *TFE3*-RCCs (24/26), 100% of *TFEB*-rearranged RCCs (19/19), and 100% of *TFEB*-amplified RCCs (17/17) [4]. Despite reporting 100% reactivity for GPNMB in *TFEB*-amplified RCCs they illustrate zonal absence of GPNMB staining in their Fig. 7E–F [4]. Li and Matoso have recently performed a retrospective analysis of GPNMB immunohistochemistry in nearly 1000 surgical pathology specimens [5]. They found that GPNMB was diffusely positive in 94.8% (218/230) of *TSC*/*mTOR*-*TFE*-related neoplasms (including RCCs with *TFE3*/*TFEB* rearrangements, PEComas, and other entities). In contrast, 83.3% of non-*TSC*/*mTOR*-*TFE*-related neoplasms were negative for GPNMB. In their study, *TFE3* or *TFEB* rearrangement, or *TFEB* amplification was identified by FISH in 46 cases. In the FISH-confirmed cases, GPNMB immunohistochemistry was positive in 40/46 tumors (87%), negative in three cases (6.5%; two with *TFEB* amplification and one with *TFE3* rearrangement), and patchy or equivocal in an additional three RCCs (6.5%; two with *TFEB* amplification and one with *TFE3* rearrangement). Overall, false-negative or equivocal GPNMB results were observed in 13% of FISH-confirmed cases, including four cases with *TFEB* amplification, and two *TFE3*-rearranged RCCs [5].

Focal or weak GPNMB staining has been also reported in other renal tumors, for example in a small subset of chromophobe RCCs, among the common renal types [3]. Some authors have thus cautioned that non-diffuse or focal GPNMB staining should be interpreted as nonspecific or negative, and only strong, diffuse cytoplasmic expression should be considered truly positive [5, 19]. However, fully recognizing the limitations of any individual stain, it is important to stress the need of appropriate use of IHC panels in the context of a given morphology and specific differential diagnosis. When considering a specific diagnosis of *TFEB*-altered RCC, one should also take into account the findings of other markers, such as Melan A and Cathepsin K, and limited/absent cytokeratin expression, particularly in limited biopsy specimens. Importantly, such IHC screening-based diagnosis requires confirmatory genomic assays, including FISH.

Our findings show that interpreting GPNMB staining in *TFEB*-amplified RCCs may be problematic when applying the criteria proposed by authors of previous studies (strong, diffuse), as a significant number of our cases (50% *TFEB*-amplified RCCs) did not exhibit diffuse GPNMB staining pattern. Consequently, we particularly recommend caution when assessing GPNMB reactivity in limited tissue samples, such as needle biopsies from renal masses. Interpretation of GPNMB immunohistochemistry can also be challenging and inconsistent when, for example, the staining is diffuse but weak, or strongly positive only in individual scattered cells [5]. Even a negative GPNMB staining does not completely exclude the diagnosis of *TFEB* amplified RCC and requires correlation with the IHC panel results and the morphology. Our study along with previous finding [5] indicate that GPNMB expression may well be affected by the specific type of molecular alteration, including differences in translocation partners, mutation sites, or underlying mechanisms (e.g., translocation vs. amplification), as well as technical issues, such as tissue fixation and antibody dilutions. Even with the limitations of its sensitivity and specificity and the variability of staining in some cases, GPNMB provides a useful and cost-effective screening tool for further diagnostic workup.

## Conclusion

We found that GPNMB was positive in all evaluated *TFEB*-altered RCCs by IHC, supporting its potential role as a sensitive screening marker. While *TFEB*-rearranged RCCs typically demonstrated strong, diffuse GPNMB immunoreactivity, *TFEB*-amplified RCCs showed more often focal or patchy staining of lower intensity. This variability highlights the need for cautious interpretation, considering the morphologic findings and the other IHC stains, particularly in limited biopsy samples. Despite these limitations, our findings support the routine use of GPNMB as an ancillary screening tool in RCC diagnostics.

## Supplementary Information

Below is the link to the electronic supplementary material.

Supplementary file1 (DOCX 22.3 KB)

Supplementary file2 (DOCX 23.1 KB)

## Data Availability

Additional data supporting study findings are available from the corresponding author on request.
