# Supplementary material for: Immunohistochemical expression of GPNMB in TFEB-rearranged and TFEB-amplified renal cell carcinomas
Source: Virchows Arch. 2026 Mar 4;489(3):695–705. doi: 10.1007/s00428-026-04466-6 (PMC13582045; doi:10.1007/s00428-026-04466-6)
Supplement: Supplementary file 1 [file 428_2026_4466_MOESM1_ESM.docx]

**Supplementary table 1.**

In group of *TFEB*-amplified RCC, only the so-called “high amplified” tumors were included. 100 randomly selected non-overlapping interphase tumor cell nuclei were analyzed for the presence of a specific positive pattern. The result interpretation of the break resp. amplification of *TFEB* gene was done using cut-off values as previously described by Skala (6).

| **Case** | **Type of the probe used for FISH** | **Number of signals** |
| --- | --- | --- |
| **Case 10**  *TFEB*-amplified RCC | ba | >15 |
| **Case 11**  *TFEB*-amplified RCC | ba | 10~15 |
| **Case 12**  *TFEB*-amplified RCC | ba | >15 |
| **Case 13**  *TFEB*-amplified RCC | ba | 10~15 |
| **Case 14**  *TFEB*-amplified RCC | ba | >15 |
| **Case 15**  *TFEB*-amplified RCC | ba | 10~15 |
| **Case 16**  *TFEB*-amplified RCC | ba | >15 |
| **Case 17**  *TFEB*-amplified RCC | enum | >15 |
| **Case 18**  *TFEB*-amplified RCC | ba | 10~15 |
| **Case 19**  *TFEB*-amplified RCC | ba | 10~15 |
| **Case 20**  *TFEB*-amplified RCC | enum | 10 |
| **Case 21**  *TFEB*-amplified RCC | enum | >15 |
| **Case 22**  *TFEB*-amplified RCC | enum | 10~15 |
| **Case 23**  *TFEB*-amplified and translocated RCC | enum | >15 |
| **Case 24**  *TFEB*-amplified RCC | enum | 10~15 |
| **Case 25**  *TFEB*-amplified RCC | enum | >15 |

*ba* - TFEB (6p21) Gene Break Apart Probe Detection Kit, *enum* - TFEB Gene Amplification Probe Reagent
