# Supplementary material for: Immunohistochemical expression of GPNMB in TFEB-rearranged and TFEB-amplified renal cell carcinomas
Source: Virchows Arch. 2026 Mar 4;489(3):695–705. doi: 10.1007/s00428-026-04466-6 (PMC13582045; doi:10.1007/s00428-026-04466-6)
Supplement: Supplementary file 2 [file 428_2026_4466_MOESM2_ESM.docx]

Supplementary table 2. Clinicopathologic data in *TFEB*-rearranged and *TFEB*-amplified RCCs

| **Case** | **Sex** | **Age** | **Dimens**  **(cm)** | **Morphology** |
| --- | --- | --- | --- | --- |
| ***TFEB*-rearranged RCC** | | | | |
| **Case 1** | f | 39 | 4.5 | acinar growth, predominantly cells with abundant clear cytoplasm and eccentric nuclei, combination with cells with finely granular eosinophilic cytoplasm, nuclear grade 2, no calcifications, no hyaline material |
| **Case 2** | f | 65 | 3 | Tubular, acinar, solid, focally cystic architecture, cells with abundant finely granular and eosinophilic cytoplasm, nuclear grade 3, no hyaline material, no calcifications |
| **Case 3** | f | 11 | 10 | Solid and cystic pattern, cells with abundant finely granular eosinophilic cytoplasm, nuclear grade 3, no hyaline material, no calcifications |
| **Case 4** | f | 63 | 5.2 | Confluent and nested growth, cells with vacuolated cytoplasm, nuclear grade 3, no calcifications, no hyaline material |
| **Case 5** | f | 67 | 6 | Solid and acinar growth, cells with abundant finely granular cytoplasm, nuclear grade 3, no calcifications, no hyaline material |
| **Case 6** | f | 14 | 5.8 | Solid growth, cells with granular and eosinophilic cytoplasm and huge clear cells with eccentric nuclei, nuclear grade 3, no calcifications, no hyaline material |
| **Case 7** | f | 27 | 10 | Solid, focally acinar, and cystic growth, cells with abundant clear cytoplasm, focally finely granular cytoplasm, nuclear grade 2, no calcifications, no hyaline material |
| **Case 8** | f | 14 | 4 | Acinar architecture, cells with abundant focally clear and focally finely granular cytoplasm, nuclear grade 3, no calcifications, no hyaline material |
| **Case 9** | m | 73 | 8.5 | Papillary and solid (confluent growth), cells with abundant granular cytoplasm, nuclear grade 3, no calcifications, no hyaline material |
| ***TFEB*-amplified RCCs** | | | | |
| **Case 10** | f | 80 | 5 | Tubulary, cystic growth, fibromyomatous stroma, cells with abundant eosinophilic cytoplasm, focally cytoplasm more scanty, nuclear grade 3, no calcifications, no hyaline material |
| **Case 11** | m | 85 | 12 | Papillary, tubulopapillary architecture, cells with abundant eosinophilic cytoplasm, nuclear grade 2, focally with transition to more clear cell areas, no calcifications |
| **Case 12** | m | 73 | 3 | Predominant confluent/solid growth, focally papillary, cystic, cells with granular eosinophilic cytoplasm, focally intracytoplasmatic vacuoles, globules, nuclear grade 3 focally 4 - bizarre, multinuclear, rhabdoid, no calcifications |
| **Case 13** | f | 74 | 8 | Solid and tubulary architecture, cells with finely granular eosinophilic cytoplasm, necrosis, nuclear grade 3, no calcifications |
| **Case 14** | m | 75 | 4.2 | Papillary, tubulopapillary structures, focally cystic and solid, abundant eosinophilic granular cytoplasm, nuclear grade 3, psammomacalcifications, fibromyomatous stroma |
| **Case 15** | m | 50 | NA | Papillary, tubulopapillary, pseudosolid, cells with abundant eosinophilic granular cytoplasm, nuclear grade 3, prominent fibromyomatous stroma, calcification |
| **Case 16** | m | 84 | 3.8 | Papillary, nested, pseudosolid, abundant eosinophilic granular cytoplasm, transition to more clear cells, nuclear grade 3, no calcifications, no hyaline material |
| **Case 17** | m | 78 | 3.5 | Focally cystic, focally tubulary/tubulopapillary to papillary growth, cells abundant eosinophilic finely granular cytoplasm, nuclear grade 3, no calcifications, no hyaline material |
| **Case 18** | m | 68 | NA | Solid and cystic growth, cells with abundant eosinophilic granular cytoplasm, nuclear grade 2, no calcifications |
| **Case 19** | f | 76 | 12 | Papillary, tubulopapillary growth, focally solid (nested), abundant finely granular cytoplasm, nuclear grade 3, no calcification, no hyaline material |
| **Case 20** | f | 72 | 3 | Papillary eosinophilic and solid clear cell areas, microcystic, nuclear grade 3, haemorrhagic and necrotic, psammomacalcifications, no hyaline material |
| **Case 21** | m | 75 | 2.2 | Solid and cystic growth, abundant eosinophilic cytoplasm, nuclear grade 3, psammomacalcifications, no hyaline material |
| **Case 22** | f | 70 | 4.5 | Papillary growth (pseudosolid focally), cells with abundant finely granular eosinophilic cytoplasm, focally cells with clear cytoplasm, nuclear grade 2, psammomacalcifications |
| **Case 23 *** | m | 74 | 13 | Solid, nested growth, cystic spaces, sporadic pseudorosettes, high-grade cells with abundant granular eosinophilic cytoplasm, nuclear grade 3, calcifications, haemorrhage |
| **Case 24** | m | 76 | 2.9 | Predominantly cystic, focally tubulary to papillary, cells with abundant eosinophilic finely granular cytoplasm, nuclear grade 2, sporadic calcification |
| **Case 25** | m | 79 | 4 | Papillary and tubulary growth, focally microcytic, cells with abundant finely granular eosinophilic cytoplasm, nuclear grade 3, reverse polarity of nuclei focally, fibrous stroma, no calcification |

m male, f female, NA not available, * *TFEB*-amplified and translocated RCC
